# Estimation of Soil Moisture from Optical and Thermal Remote Sensing: A Review

**DOI:** 10.3390/s16081308

**Published:** 2016-08-17

**Authors:** Dianjun Zhang, Guoqing Zhou

**Affiliations:** 1 The Center for Remote Sensing, Tianjin University, Tianjin 300072, China; zhangdianjun123@163.com; 2 College of Earth Sciences, Guilin University of Technology, Guilin 541004, China; 3 Guangxi Key Laboratory for Spatial Information and Geomatics, Guilin University of Technology, Guilin 541004, China

**Keywords:** soil moisture (SM), optical and thermal remote sensing, vegetation index, land surface temperature

## Abstract

As an important parameter in recent and numerous environmental studies, soil moisture (SM) influences the exchange of water and energy at the interface between the land surface and atmosphere. Accurate estimate of the spatio-temporal variations of SM is critical for numerous large-scale terrestrial studies. Although microwave remote sensing provides many algorithms to obtain SM at large scale, such as SMOS and SMAP etc., resulting in many data products, they are almost low resolution and not applicable in small catchment or field scale. Estimations of SM from optical and thermal remote sensing have been studied for many years and significant progress has been made. In contrast to previous reviews, this paper presents a new, comprehensive and systematic review of using optical and thermal remote sensing for estimating SM. The physical basis and status of the estimation methods are analyzed and summarized in detail. The most important and latest advances in soil moisture estimation using temporal information have been shown in this paper. SM estimation from optical and thermal remote sensing mainly depends on the relationship between SM and the surface reflectance or vegetation index. The thermal infrared remote sensing methods uses the relationship between SM and the surface temperature or variations of surface temperature/vegetation index. These approaches often have complex derivation processes and many approximations. Therefore, combinations of optical and thermal infrared remotely sensed data can provide more valuable information for SM estimation. Moreover, the advantages and weaknesses of different approaches are compared and applicable conditions as well as key issues in current soil moisture estimation algorithms are discussed. Finally, key problems and suggested solutions are proposed for future research.

## 1. Introduction

Soil moisture (SM) is an important variable in land surface system research, including studies of the regional water cycle, agricultural irrigation management, climate change and environmental monitoring [1,2,3,4,5,6,7]. SM links the energy and water exchange between the atmosphere and land surface and indicates groundwater conditions [8,9]. Additionally, SM is used to indicate drought in vegetated environments because water content is an essential factor for vegetation growth [10,11,12]. This means that SM plays an important role in the terrestrial water cycle [13,14,15,16]. When soil moisture evaporates into the atmosphere, energy exchange occurs between the atmosphere and land surface. On the other hand, surface water and groundwater are linked with the vertical migration of the SM that is taken up by vegetation for survival. Because the SM plays two vital roles, it is an indispensable variable in terrestrial research. SM estimation studies originated in the 1960s because of the limitations of measurement equipment and theoretical immaturity; however, this research mainly relies on empirical relationships and point-based experiments [17,18,19]. In the 1970s and 1980s (corresponding with theoretical advances), SM was treated as an important parameter in conjunction with other surface parameters in comprehensive studies [20,21,22,23,24,25,26]. Since the 1990s, several studies have been performed to determine methods for estimating SM [27,28,29,30,31,32]. In addition, due to the advent of remote sensing technologies, researchers have gradually shifted their attention to monitoring large areas by using remotely sensed data. Based on the optical and thermal infrared remote sensing, many approaches have been developed by establishing relationships between SM and soil reflectivity or surface temperature/vegetation coverage and soil thermal properties. Based on the microwave remote sensing, various methods have been proposed for the past 35 years [6,33]. For passive microwave remote sensing, the representative models are GOM [34], POM [34], SPM [34], IEM [35,36], AIEM [37,38], Q/H [39,40] and Q/P [41] for bare soil area. The representative soil moisture estimation models for vegetation area are MIMICS model [42], water-cloud model [43], crop model [44] and f-k model [45,46]. Due to the close relationship between backscatter coefficients and SM, researchers developed different SM modeling efforts from active microwave sensors, such as Empirical models [47,48], Semi-empirical models [49,50,51] and Physically-based models [52,53,54]. At the regional scale, SM has been obtained by using remote sensing techniques, which represent a new era in soil moisture estimations.

Traditional SM observations mainly include single-point based or specific location measurements [55,56,57,58,59]. The direct and most accurate method is the gravimetric method [60]. The original soil moisture content can be determined from changes in mass. However, this method is destructive and cannot be reproduced. Moreover, this method requires a large amount of manpower for sampling and lab measurements [61]. Time domain reflectometry (TDR), neutron probes and gamma ray scanners are subsequently used to indirectly measure the SM. These methods are point-based and cannot be used as a proxy for the regional SM.

The development of satellite technology provides a powerful method for monitoring the spatial-temporal variations of regional SM and provides quantitative estimations. Many experts have established different methods for retrieving SM based on the relationships between the SM and satellite-derived land surface parameters. Using the visible infrared band, the SM can be derived from the relationships between soil reflectance and different SMs or vegetation traits that occur under water stress. These methods can be divided into two categories, (1) the single spectral analysis method; and (2) the vegetation index method. For thermal infrared wavebands, the parameters related to the soil thermal properties are mainly used to derive the SM. This method includes two categories, (1) the thermal inertia method; and (2) the temperature index method. Combining visible and thermal infrared remotely sensed data can provide more information for estimating soil moisture than the single one. Spatial and temporal information-based methods have their own advantages and disadvantages. It is therefore important to determine how to combine these methods reasonably to obtain highly accurate SM.

In this article, SM estimation methods relying on optical and thermal remote sensing are collated and summarized. Starting from the main methods currently used, the SM estimation status and major issues are introduced. Next, the physical principles, advantages and existing problems regarding the different methods are presented in detail. Finally, several recommendations are made based on current issues faced when estimating SM. The overall framework of different remote sensing methods used in soil moisture estimation is summarized in Table 1.

## 2. SM Estimation from Optical and Thermal Remote Sensing

Although the signal penetration capabilities of optical and thermal methods are worse than those of thermal infrared and microwave methods, the reflectance domain is the most operational because the images are easily available over a broad range of ground resolutions [77,78,79,80,81,82]. Wavelengths of 0.4–2.5 μm represent the radiation of the sun reflected from the Earth’s surface [83,84]. These approaches mainly use reflectance to estimate the SM in visible infrared remote sensing techniques. In this paper, according to the different characteristics used of many bands in visible infrared remote sensing, they are divided into the single spectral analysis method and vegetation index method.

### 2.1. Single Spectral Analysis Method

The influences of soil moisture on spectral reflectance were observed in early studies [85,86]. The reflectance of water absorption bands is different from that of non-absorption bands. The absorption amplitude and presented reflectance differences are used to establish relationships with soil moisture. From the experimental results on many soils with different SMs as early as 1925, reflectance has been observed to decrease as the SM increases [85]. Since then, many scientists have reproduced this phenomenon and have established empirical relationships between soil reflectance and SM [87,88]. In 1972, Bowers and Smith demonstrated that the absorption amplitude was linearly related to the SM at the water absorption bands [86]. Based on experimental results, Jackson et al. [89] found that the albedos of all dry soils were approximately two times greater than those of wet soils, except for sand. By analyzing the water absorption values measured in the near-infrared band, Dalal et al. [90] accurately estimated the SMs of a large number of soil samples. However, the above-mentioned methods are all limited to specific regions. Because of the different physical compositions, soil textures and surface roughness in different study areas, empirical equations cannot be applied universally.

Corresponding with the development of monitoring instruments and theoretical advances for estimating soil moisture, Jaquemoud et al. [91] developed the SOILSPEC transmission model, which considers soil roughness, viewing angle and the inherent spectral characteristics of soil to obtain the bidirectional reflectance of the soil from 450 nm to 2450 nm. Later, Lobell et al. [92] established a physical model and indicated that an exponential relationship exists between soil reflectance and SM by analyzing the changes in the reflectance of four different soils at various moisture contents. The model can be expressed simply as

(1) $$R=f\times R_{dry}+(1-f)\times R_{dry}\times (-c\times s)$$

where *R_dry_* is the dry soil reflectance, *c* describes the change rate induced by SM, *s* is the soil saturation, and *f* is the saturation rate.

Liu et al. [93] applied a simple stepwise linear regression method to reduce the band numbers and found that the soil reflectance decreased as the SM increased when the soil moisture was low. However, after the soil moisture reaches the critical point, the reflectivity increases as the SM increases. This critical point is closely related with the soil hydrodynamic characteristics. Based on previous studies, Liu et al. [94] evaluated three soil reflectance models using 18 soils and the following methods: the relative reflectivity method, the derivative/difference method and the discrete band difference method. The most applicable method is the derivative/difference method because it can be used to estimate the SM precisely and is less sensitive to external factors, such as the atmosphere and the large variation of soil roughness and soil types, etc. Similarly, Whiting et al. [95] obtained better results by using the Gaussian model to estimate the SM. Using a linear mixed pixel separation algorithm in the near-infrared—red band space, Gao et al. [96] used soil line equations and empirical formulas to derive the soil reflectance of mixed pixels under vegetation cover. Combined with the TM images for field verification, this method is applicable at different spatial-temporal scales and the results are credible.

Based on laboratory measurements of soil spectral information, single spectral analysis approaches are generally used to determine the relationships between SM and soil reflectance that can obtain reasonable results for specific soil samples. However, in practice, soil reflectance is affected by many factors, such as vegetation, the atmosphere, etc. The measured satellite signals on the surface will be influenced or reduced, affecting the reflectance and its operability. In addition, such methods are statistical and empirical models that lack physical basis to quantitatively determine the SM.

### 2.2. Vegetation Index Method

Because it is extremely sensitive to water stress, the vegetation index estimated by remote sensing has been widely used to detect drought conditions, such as the normalized difference vegetation index (NDVI) and enhanced vegetation index (EVI) [97]. Drought conditions can be reflected by vegetation growth, which can be directly represented by the vegetation index. By comparing the vegetation index at different times, the impacts of drought can easily be determined. However, the accuracy of using NDVI-based drought indices is often subject to changes in vegetation types and ecosystem differences. Additionally, the NDVI-based drought index may be a conservative indicator [32] because droughts caused by water stress or rainfall anomalies will both have time delays [98]. A detailed overview of the drought indices for regional monitoring was provided by Richard and Heim [99].

Many classical drought indices based on vegetation indicators were proposed and have been widely used in drought monitoring. To remove the influences of weather and geographic location differences on NDVI, Kogan [100,101] developed the following Vegetation Condition Index (VCI) based on statistical NDVI time-series data:

(2) $${VCI}_{ij}=\frac{100*({NDVI}_{ij}-NDVI-MIN_{j})}{NDVI-MAX_{j}-NDVI-MIN_{j}}$$

where VCI is a modified NDVI, vegetation condition index; NDVI is a weekly smoothed composite Normalized Difference Vegetation Index. NDVI-MIN and NDVI-MAX are the maximum and minimum values of NDVI, respectively, which can be obtained from historical data. *i* and *j* denote weeks and locations. VCI not only describes the surface vegetation cover and spatial-temporal variations but also quantifies the impacts of weather on vegetation. Furthermore, VCI can be used to evaluate the effects of weather on different ecosystems.

Considering the error caused by the instantaneous vegetation index and using the multi-year average NDVI values as criterion, the difference in amplitude was calculated from the NDVI values obtained over one month or ten days minus the reference value, which are positive or negative anomalies of the vegetation index. Chen et al. [102] developed an anomaly vegetation index (AVI) to study annual vegetation dynamics.

The monthly AVI is calculated as follows:

(3) $$AMTNDVI=(MTNDVI-\bar{MTNDVI})$$

where *AMTNDVI* is the anomaly vegetation index, $\bar{MTNDVI}$ is the average value for the same month in different years, and *MTNDVI* is the monthly vegetation index for a specific year.

**Figure 1:**
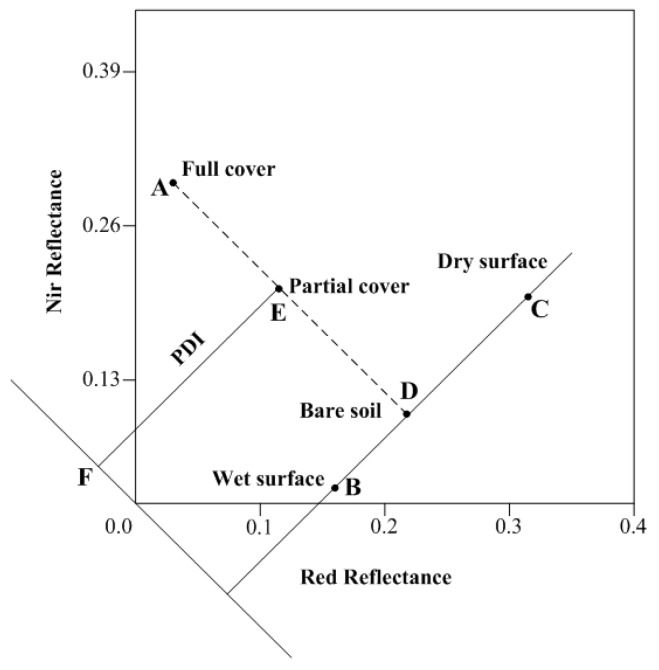
Near Infrared–red space and Perpendicular Drought Index [107].

Because its sensitivity to water differs, the relationships among different water absorption bands were applied to derive the SM. Gao et al. [103] presented a normalized difference water index (NDWI) using the reflectance at 1.24 µm rather than 0.66 µm because the former is more sensitive to SM. More importantly, the NDWI is insensitive to the atmospheric conditions.

(4) $$NDWI=\frac{\rho \;\left(0.86\;\mu m\right)-\rho \;\left(1.24\;\mu m\right)}{\rho \;\left(0.86\;\mu m\right)+\rho \;\left(1.24\;\mu m\right)}$$

where *p* is the reflectance. Based on the *NDWI*, Wang et al. [104] proposed using the normalized multi-band drought index (*NMDI*), which uses the band differences between the sensitivity of 1640 nm and 2130 nm for soil and vegetation, respectively, with enhanced sensitivity to drought monitoring. The successful application in detecting forest fires demonstrated that NMDI can provide a quick response to moisture changes [105,106].

(5) $$NMDI=\frac{R_{860\;nm}-(R_{1640\;nm}-R_{2130\;nm})}{R_{860\;nm}+(R_{1640\;nm}-R_{2130\;nm})}$$

where *R* is the reflectance. Considering the effects on the spectral signatures of soil moisture variations, a new method was developed for drought monitoring [107]. Based on the spectral pattern of SM changes in the near-infrared–red band space, the Perpendicular Drought Index (PDI) was established using ETM reflectance (Figure 1).

For a specific soil type, the soil line can be regarded as a line that characterizes the spectral behavior of non-vegetated pixels when the soil moisture content varies noticeably [108]. The PDI describes the SM distribution in the near-infrared—red space. The points farther away from the coordinate origin indicate low soil moisture areas. In contrast, the closer points represent wet conditions and the PDI is expressed as follows:

(6) $$PDI=\frac{1}{\sqrt{M^{2}+1}}(R_{Red}+MR_{NIR})$$

where *R_NIR_* and *R_Red_* denote the reflectance of near-infrared and red band, respectively; M represents the slope of the soil line. The *PDI* is suitable for bare soil but has many limitations in vegetated areas, especially for non-flat regions with different soil types.

Ghulam et al. [108] introduced fractional vegetation coverage (FVC) for the removal of vegetation information from mixed pixels and proposed the Modified Perpendicular Drought Index—MPDI. If the relationship between the reflectance of vegetation and bare soil is linear, the MPDI can be expressed as follows:

(7) $$MPDI=\frac{R_{Red}+MR_{NIR}-f_{v}(R_{v,Red}+MR_{v,NIR})}{(1-f_{v})\sqrt{M^{2}+1}}$$

where *R_v,Red_* and *R_v,NIR_* are the vegetation reflectances in the red and near-infrared band, respectively, generally set to 0.05 and 0.5 by field measurements [108]. *f_v_* is the vegetation cover. By considering the SM status and vegetation growth, the MPDI can be applied to vegetated regions to obtain credible results.

The vegetation indicator method can reflect the SM conditions from the vegetation index changes. The advantages and disadvantages of these commonly used drought indices are described in Table 2. Because the vegetation index cannot immediately reveal information when the vegetation is restricted by water stress, a long period is required to show the water-limited features after some accumulation; thus, this method requires a certain time lag to show real-time SM conditions. Second, the inter-annual changes of land cover types may affect drought monitoring results. Therefore, interpretation of the monitoring results should require the latest reliable land cover map. Simultaneously, these methods only consider that water stress leads to reductions in the NDVI and do not account for other factors, such as changes in temperature and rainfall. In addition, good quality satellite data obtained over a long period of many years must be obtained, and the obtained results only reflect the SM deficit situation and not the true soil moisture content.

## 3. SM Estimations from Thermal Infrared Remote Sensing

In many surface temperature studies, the surface temperature variations at the thermal wavebands or the soil thermal properties were strongly correlated with the SM. Due to the special thermal properties of the soil in the thermal infrared band and its theoretical maturity, SM estimations are gradually achieved. The thermal inertia and temperature index methods are two main methods with their own advantages and disadvantages. The development process and their advantages and limitations are introduced in the following. Also, some useful references and recommendations were provided.

### 3.1. Thermal Inertia Method

As a property of the substance itself, thermal inertia is often used for mineral exploration, lithological mapping and geological research. Thermal inertia was highly correlated with SM in numerous laboratory experiments and is defined as the resistance to temperature variations that is induced by exterior energy and controls the amplitude of the temperature change. The higher the thermal inertia, the less the temperature varies. The relationship between thermal inertia and SM can be determined quantitatively from the changes in soil temperature or the diurnal amplitude of the surface temperature. Generally, thermal inertia includes the soil thermal conductivity and soil heat capacity. The soil thermal inertia can be expressed as follows:

(8) $$P=\sqrt{\lambda \rho C}$$

where *λ* is the soil thermal conductivity, *ρ* is the soil bulk density, and *C* is the soil heat capacity. When the SM increases, the thermal inertia increases correspondingly and reduces the diurnal amplitude variations of the land surface temperature (LST).

#### 3.1.1. The Physical Analytical Model

In geophysical research, soil thermal properties and SM show certain relationships [109,110]. Watson [26] first proposed physical analytical equations of thermal inertia that were retrieved from remotely sensed data. The SM was successfully estimated using several meteorological elements, the soil moisture profile and remote sensing information. In addition, Kahle [23] conducted many studies on thermal inertia models, proposed different approaches for solving one-dimensional heat conduction equations, and attempted to integrate remotely sensed data to estimate thermal inertia for large regions. In addition, Price [111] simplified the latent and sensible heat flux expression and combined the surface one-dimensional heat conduction equation with the energy balance principle by using Fourier transformation to introduce a comprehensive surface parameter B (B is a function of soil emissivity, air humidity, temperature and other meteorological factors). Because of the requirements of a large number of observed ground data in the B solving process, this approach is unavailable for many regions.

Many thermal inertia models have treated thermal rock and soil characteristics as a simplified linear function of temperature [23,26]. However, Engman [112] considered that this relationship is nonlinear and includes factors such as the effects of night frost. When combined with microwave data to solve the thermal inertia in vegetated areas and validate in situ measurements, the obtained results were reasonable [113].

Based on the above-mentioned models, Price [111] proposed using the classical apparent thermal inertia (ATI) by removing the latent heat of evaporation. The ATI can be expressed as follows:

(9) $$ATI=1000\pi \frac{\left(1-\alpha \right)\cdot C_{1}}{T\left(1:30\;p.m.\right)-T\left(2:30\;a.m.\right)}$$

where 1000*π* is used to make the ATI range from 0 to 255. *C*_1_ is the constant term. *T* is the land surface temperature, and α is the surface albedo.

The ATI model is accepted by many scholars, and extensive studies have been conducted in different areas. Combined with the ATI model and MODIS data, Stephen et al. [114] quantitatively estimated the SM and sediment availability for a White ShashaQiu field in New Mexico. Verstraeten et al. [115] used the ATI model to obtain the SM conditions in Xinjiang and the soil moisture content by using a two-source water balance model at a depth of 1 m. The results were verified by using TDR measurements, and the drought distribution and trends were analyzed in Xinjiang. Frank et al. [116] fit the diurnal temperature variation curve of the in-situ measured surface temperature in combination with the albedo to obtain the ATI and used AMSR-E soil moisture data for validation. The greatest advantage of this method is that surface temperature can be obtained from the observation sites, which is beneficial for the equatorial regions. Chang et al. [117] studied the soil moisture conditions of different types of land in tropical mountains using the ATI model.

To accurately derive the SM, Chen et al. [118] monitored the spring drought by using the improved ATI model for the Hebei Plain. Next, 1 km pure pixels were merged with images with 30 m resolution from the 1st environmental satellite (HJ-1) and the drought was accurately monitored in the vegetated mixing zone. It was concluded that the traditional ATI model was the most suitable for bare land areas and that the inversion accuracy of the mixed pixels was poor.

The physical thermal inertia model can provide information regarding hydrothermally relevant principles and the relationships with SM; however, this model requires detailed atmospheric forcing data inputs and a complex calculation process. ATI is used as a proxy of thermal inertia, and because many factors are truncated, accuracy is often not guaranteed. In addition, ATI is not applicable in vegetated regions. For vegetated areas, FVC should be used to estimate thermal inertia. Moreover, the ATI can be combined with other soil moisture indexes to calculate the SM [115]. Because ATI is simple and easy to operate, it is promising for broad applications.

#### 3.1.2. The Model Based on the Amplitude and Phase Information of LST

Xue and Cracknell [119] introduced land surface temperature phase information to simplify the analytical thermal inertia model, in which only the ground auxiliary data, the time that the maximum LST occurs, is required and the real thermal inertia can be obtained. This approach greatly simplifies the sophisticated calculation process. Thus, many scholars have subsequently estimated SM using this method. Cai et al. [120] used the full thermal inertia model of [119] and a second order approximation approach to retrieve the thermal inertia surface that was applied to north China to monitor the SM status and achieve good results.

The main equation of the full model [121] is

(10) $$P=\frac{(1-A)S_{0}C_{t}}{\Delta T\sqrt{\omega }}\frac{A1[\cos(\omega t_{2}-\delta_{1})-\cos(\omega t_{1}-\delta_{1})]}{\sqrt{1+\frac{1}{b}+\frac{1}{2b^{2}}}}\delta_{1}=\omega t_{\text{max}}-2m\pi ,m=0,1,2\ldots$$

where *A* is the albedo, *S_0_* is the solar constant. *b* is a synthesized term. *C_t_* is the atmospheric transmittance in the visible spectrum (typically 0.75). *w* is the Earth rotation velocity. *δ* is the phase angle. ΔT is the temperature difference. If *n* = 1, *P* can reach a maximum value. In addition, *t* is the time at which the maximum LST occurs.

The model proposed by Xue and Cracknell requires important auxiliary parameters from the ground-based observations (the time of the maximum LST occurs) but, in practical applications, the maximum air temperature observation time is used as an input value. Because the maximum LST and air temperature occurred differently with time, the model induces uncertainties by using the maximum air temperature. Because polar-orbiting satellites are limited by the times that they pass over the same area and cannot obtain constant observations, the method proposed by Sobrino includes a certain degree of difficulty. However, the emergence of geostationary satellites would support this method and many observations can be obtained for the same study area in one day. In addition, the use of observation periods in the model has not been specifically validated.

Based on the model proposed by Xue and Cracknell, Sobrino et al. [122,123] developed the Four Temperature Algorithm (FTA) to calculate soil thermal inertia by using three surface temperatures at different times on the same day from all the calibrated remote sensing results, including viewing geometry and atmospheric correction. The results were reasonable and verified for the Iberian Peninsula and Morocco.

The key process of the FTA model is:

(11) $$\begin{array}{c} \delta_{1}=\text{arctan}(\xi )+(2m\pi +1),m=0,1,2\ldots \\ \xi =\frac{\left(T_{j}-T_{k})[\cos(\omega t_{i})-\cos(\omega t_{j})]-(T_{i}-T_{j})[\cos(\omega t_{j})-\cos(\omega t_{k})\right]}{\left(T_{i}-T_{j})[\sin(\omega t_{j})-\sin(\omega t_{k})]-(T_{j}-T_{k})[\sin(\omega t_{i})-\sin(\omega t_{j})\right]} \end{array}$$

where the symbols in the above equation is the same as the Equation (10). This method used three different time phases to solve the equations, and the phase angle information can be written as a function of LST and satellite overpass time, which eliminates the ground auxiliary data; thus, the soil thermal inertia can only be obtained from remotely sensed data. Because it is difficult to obtain more than two high-quality remotely sensed images in one day from remote sensing satellites (except geostationary satellites) and because of night clouds and atmospheric effects, this method has encountered many problems in practical applications and is difficult to use universally.

Recently, people actively sought high-accurate methods for solving thermal inertia. Wang [106] proposed using a simple thermal inertia estimation method based on idealized soil temperature to obtain an analytical solution for diffusion in the sinusoidal form. Thermal inertia can be expressed as a scale factor in a linear relationship between the soil heat flux and surface temperature amplitude; thus, the estimation accuracy was improved. The biggest advantage of this method is that the available input parameters can be easily obtained from remotely sensed data. Moreover, fewer model input parameters are needed and the precision is high.

Maltese [124] analyzed the applicable conditions of a thermal inertia model in sparsely vegetated areas and introduced a correction factor for cloud effects to rectify downward shortwave radiation. The applicability of the thermal inertia model was evaluated under changeable weather conditions. Meanwhile, Robock et al. [125] studied the data quality of a thermal inertia model combined with a two-source model to separate skin temperature for vegetation and bare soil and conducted a sensitivity analysis of the input parameters, such as solar radiation and meteorological data. The thermal inertia was obtained by using thermal budget model.

#### 3.1.3. Analysis Method Based on Energy Sources

Verhoef [126] indicated that the direct energy source of thermal inertia is soil heat flux rather than net radiation and developed a surface temperature difference method between sunset and sunrise for solving thermal inertia. If the latent heat flux and sensible heat flux are both zero and the net radiation remains constant on clear windless nights, the following formula can be used:

(12) $${\left(C_{h}\sqrt{D_{h}}\right)}_{r}=\frac{2\left|\bar{Rn}\right|\sqrt{\Delta t}}{\Delta T_{s}\sqrt{\pi }}$$

where $\left|\bar{Rn}\right|$ is the average net radiation flux between sunset and sunrise (W·m^−2^), Δt is the time interval (s), and $\Delta T_{s}$ is the decrease in LST between sunset and sunrise (°C), C_h_ is the soil heat capacity (MJ·m^−3^·K^−1^), D_h_ is the thermal diffusivity (m^2^·s^−1^).

By comparing different methods to estimate thermal inertia and soil heat flux, Murray and Verhoef [127,128,129] confirmed that this approach is relatively simple and reliable. However, because of the harsh applicable conditions, this method has rarely been used. First, this method requires a clear and windless night with constant net radiation. These weather conditions are generally difficult to satisfy. In addition, it is difficult to guarantee image quality at night, which results in incorrect estimation results.

#### 3.1.4. Remote Sensing Methods Combined with Soil Physical Parameters

A relationship with soil moisture can be established based on the definition of thermal inertia if soil characteristics and remotely sensed data are available for estimating thermal inertia. Lu et al. [130] proposed using a simple model that considers the soil solid composition and other known pivotal soil characteristics, such as soil composition and density.

The main equations are calculated as follows:

(13) $$P=P_{dry}+(P_{sat}-P_{dry})K_{p}$$

(14) $$K_{p}=\text{exp}[\gamma (1-S_{r}^{\gamma -\delta })]$$

where *P_dry_* and *P_sat_* are the dry and wet soil thermal inertia, respectively, and *K_p_* is the Kersten correction function. *γ* and *δ* were soil texture dependent model parameters and *S_r_* was the degree of saturation.

In addition, Verstraeten et al. [106] used the apparent thermal inertia and soil moisture saturation index (SMSI) combined with Meteosat Second Generation (MSG) data to estimate the SM. When verifying the analysis by using in-situ data and the microwave moisture content, the results showed that the values and trends fit well. M. Minacapilli [131,132] normalized the *K_p_* function by using ATI with remotely sensed data to obtain the actual thermal inertia based on the soil porosity.

Thermal inertia methods focus on soil thermal characteristics and have clear physical meanings that are relatively easy to understand. Different methods are compared in Table 3. In practical applications, clear day and night images are difficult to obtain for the same area of interest because of clouds, and the accuracy for night image is difficult to confirm. Simultaneously, the assumption that the soil properties at the horizontal and vertical scale are consistent is not readily satisfied. Moreover, in areas with high vegetation cover, vegetation conceals the soil information, which affects the estimation accuracy. Thus, this method is only applicable to bare and sparsely vegetated areas.

### 3.2. Temperature Index Method

Land surface temperature is one control parameter for the physical and biological processes that occur at the land surface. For bare soil area, LST refers to the soil surface temperature; and for densely vegetated areas, the LST indicates the vegetation canopy temperature under the assumption of energy balance. An increase in the vegetation canopy temperature is an initial indicator that the vegetation is subject to water stress. Therefore, the LST can be used to monitor the SM.

#### 3.2.1. Normalized Difference Temperature Index

Considering the seasonal variations of the LST, Mcvicar et al. [133] developed the normalized difference temperature index (NDTI) to reflect the SM conditions. The NDTI is calculated as follows:

(15) $$NDTI=\left(LST_{\infty }-LST\right)/\left(LST_{\infty }-LST_{0}\right)$$

where $LST_{\infty }$ and $LST_{0}$ are the simulated LSTs of the infinite and zero surface impedance, respectively, which are the upper (extremely dry state) and lower (extremely wet state) boundary conditions for the LST at specific atmospheric forcing and surface impedance. The surface energy balance principle and aerodynamic impedance model can be used to obtain the extreme LSTs.

NDTI eliminates the effects of the seasonal variations of LST. The SM is very similar to the NDTI [134]. Thus, the NDTI can accurately reflect the spatial-temporal variations of SM. However, this model requires solar radiation, wind speed and LAI as input values, which could limit the operability of the model.

#### 3.2.2. Crop Water Stress Index

Soil water availability is a basic variable for the evapotranspiration process. In addition, the SM affects the evapotranspiration rate. Evapotranspiration and energy are closely related to the SM. When the energy is high and the SM is sufficient, strong evapotranspiration occurs and the canopy temperature is lower. By contrast, evapotranspiration is weak and the canopy temperature increases when the vegetation is subjected to water stress. For a specific atmospheric condition, the ratio of actual and potential evapotranspiration can be used as a proxy for crop water stress. Based on the surface energy balance model, Jackson et al. [135] proposed using the crop water stress index (CWSI) to mirror the ratio between vegetation transpiration and maximum potential evaporation. The CWSI is obtained from the vegetation canopy temperature, ambient air temperature and net solar radiation. The CWSI formula is shown as follows:

(16) $$CWSI=\frac{(Tc-Ta)-{\left(Tc-Ta\right)}_{\text{min}}}{{\left(Tc-Ta\right)}_{\text{max}}-{\left(Tc-Ta\right)}_{\text{min}}}$$

where *Tc* is the vegetation canopy temperature, *Ta* is the air temperature, and (*Tc–Ta*)_max_ and (*Tc–Ta*)_min_ are the difference between the canopy and air temperature without transpiration and potential evaporation, respectively.

The CWSI uses thermal infrared temperatures and meteorological data to indirectly reflect the SM under crops. This method has clear physical meaning based on the surface energy balance principle. In addition, this method is highly precise in regions covered by vegetation and the estimation accuracy is higher than that of the thermal inertia method [136]. However, the CWSI was established based on the single canopy energy balance model, which is less effective for early crop growth. In addition, this approach requires meteorological data and the calculation process is complex. Furthermore, the extrapolation methods used for meteorological data, which are mainly obtained from ground weather stations, have important impacts on the accuracies of the CWSI determinations.

## 4. SM Estimations from Visible and Thermal Infrared Remote Sensing Data

### 4.1. The Spatial Information-Based Method

Many studies have demonstrated that LST and VI (vegetation index/vegetation coverage) can provide vegetation water stress conditions and SM information [137,138,139,140,141,142,143,144,145,146,147,148]. If the number of pixels in the study area is sufficient and the clouds and water bodies are removed, the spatial distribution of LST and VI constitute triangular or trapezoidal feature space. The SM can be obtained from the pixel distribution in the LST-VI feature space.

Because the surface energy maintains balance in the LST-VI feature space, lower latent heat flux results in more available energy for the surface sensible heat flux. For transpiration, which is a crucial parameter, the stomatal resistance is influenced by the soil moisture availability. In sparsely vegetated regions, the thermal soil inertia affects the LST because it affects the transfer of heat into the soil and the soil heat flux [149,150]. These thermal characteristics are a function of soil type and soil moisture variations [151]. In addition, the surface available energy (Rn-G) also influences the LST. The control of the surface temperature by radiation means that low net shortwave radiation areas have lower temperatures. In addition, the surface albedo is affected by soil type, soil moisture and vegetation cover. Furthermore, incident radiation affects transpiration and stomatal resistance, and the net radiation can be decomposed to sensible and latent heat flux. The transfer of energy as heat from the surface to the atmosphere is an important component for surface temperature and explains why the temperatures in areas with high vegetation coverage roughness are lower than those in areas with bare soil, which influences the shape of the LST-VI space.

The LST-VI feature space is restricted by the dry and wet edges. The wet edge in the feature space represents maximum evapotranspiration and adequate soil moisture and the dry edge indicates that the vegetation is subjected to water stress in which evapotranspiration reaches the minimum and the SM is 0 (no water can be used). For different vegetation types, the dry edge includes the driest points and the vegetation evapotranspiration varies depending on the amount of vegetation coverage. For certain types, the available energy (Rn-G) is considered nearly equal. Because cooling affects evaporation, the surface temperature is maximized at the dry edge and minimized at the wet edge. Different views of the surface temperature in the fully vegetated area resulted in the development of the triangular and trapezoidal methods. The triangular method assumes that the surface temperatures of fully vegetated areas are equal to the wettest surface temperature. However, the trapezoidal method assumes that the surface temperatures of fully vegetated surfaces are much higher than those of the wettest surface.

#### 4.1.1. The Triangle Method

**Figure 2:**
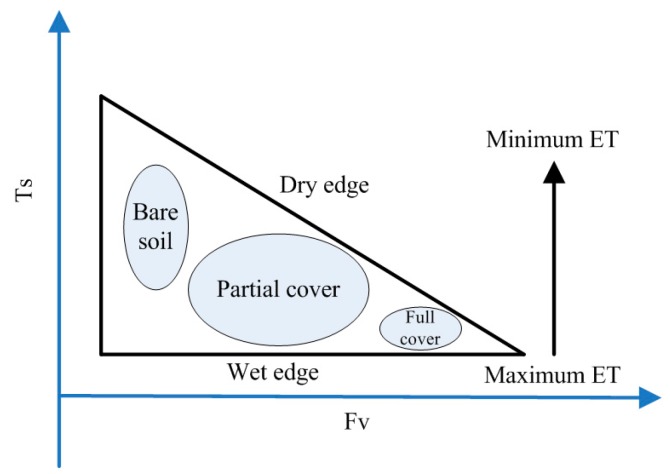
The idealized triangle space between Ts and Fv [138].

With the feature space constructed by LST and VI, Price [152] first proposed the concept of triangular space. The triangle method is proposed based on surface temperatures and VI scatter plots obtained using satellites (Figure 2). Combined with the simulation results of the land surface model, the simulated feature space is stretched to the satellite by analyzing the position of the image element in space to infer the SM.

The LST-VI triangular feature space established under the full ranges of SM and vegetation is characteristic of a space bounded with an upper decreasing envelope and a lower nearly horizontal envelope with increasing vegetation cover and with two envelopes ultimately intersecting at a (truncated) point of full vegetation cover [153]. Price [152] presented the LST-VI triangular space using satellite remotely sensed AVHRR data and analyzed the impacts of cirrus on soil moisture. Nemani [147] studied the scale effects of the LST-VI space and its orographic influences on LST and VI and indicated that vegetation type and topography are two decisive factors. Similarly, Clark [142] applied multi-spectral data to monitor drought and presented an empirical model. Mallick [154] retrieved SM using the triangle method at the field and landscapes scales by using ASTER and MODIS, respectively. Both results were reasonable. The inversion accuracy is much lower for high spatial resolution images because it is more difficult to reflect heterogeneity within the pixels.

The main assumptions of the triangle method are that (1) the complete underlying vegetation cover from the bare soil to full vegetation is know; (2) the variations in the space are not primarily caused by differences in atmospheric conditions but are caused by variations in the available SM; and (3) the sensitivity of LST to the canopy and soil is different. Due to the special characteristic of triangular space, the major advantages of this method include the following: (1) it can estimate the SM from entirely remotely sensed data without any ground auxiliary data; and (2) it is simple and easy to operate and can monitor drought conditions over large regions. Meanwhile, the limitations include the following: (1) the LST-VI space determination requires a certain degree of subjectivity; (2) the number of pixels is large enough to span a wide range of soil wetness and vegetation cover in the study area; and (3) many limitations occur in regions that are not flat.

#### 4.1.2. The Trapezoid Method

Using experiments, Jackson [155] explored the relationships between drought and differences in vegetation leaf and air temperature by using measured data and proposed a drought index—CWSI. To calculate the pixel values of intermediate vegetation cover and SM for a specific time, four vertices of the trapezoid are required that correspond to (1) well-watered, full-cover vegetation; (2) water-stressed, full-cover vegetation; (3) saturated bare soil; and (4) dry bare soil. These values should be computed first by using the CWSI theory and Penman-Monteith equation. This index can only be used for fully vegetated areas and is not applicable in sparsely vegetated areas.

**Figure 3:**
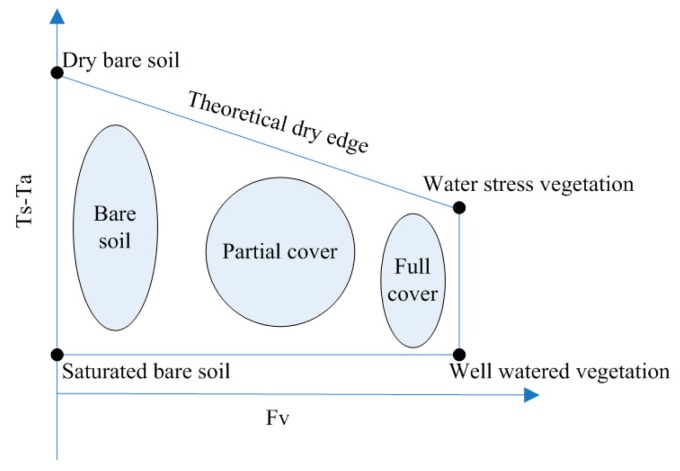
The simplified trapezoidal space between Ts-Ta and Fr [31].

Based on the theory of the CWSI, Moran [31] developed the water deficit index (WDI) (Figure 3) to overcome the above-mentioned shortcomings. This index can be used in all areas covered by vegetation and in areas with sparse vegetation. Relying on the surface energy balance principle, the soil adjusted vegetation index (SAVI) and the temperature difference form a trapezoidal space, and the index can be directly calculated from remotely sensed data without requirements for measuring leaf and air temperature. After the model test and in situ data validation, the WDI was evaluated to overestimate the water deficit situations in many sparsely vegetated regions.

Compared with the triangular space, the trapezoidal space does not require a large number of pixels. The advantages of the trapezoidal method are that (1) it has a robust physical basis and that (2) the space determined from the four vertices is the main limiting condition that is close to the true conditions of the land surface. In contrast, the disadvantages include that this method (1) requires more ground-based parameters to calculate the soil moisture index and that (2) the influences of water stress on vegetation cover have a time lag that induces uncertainties for SM estimations.

### 4.2. The Temporal Information-Based Method

**Figure 4:**
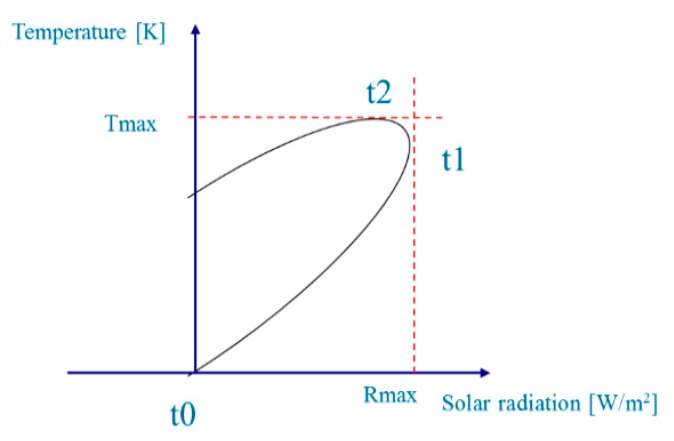
The relationship between the land surface temperature and net surface shortwave radiation.

Polar-orbiting satellites can only provide several remotely sensed data points each day, which does not satisfy the requirements of long time series data. However, geostationary satellites can provide more data than the polar-orbiting ones. Based on the advantages of geostationary satellites, net surface shortwave radiation (NSSR) and LST form an elliptical shape [156,157] (Figure 4). By conducting a systematic sensitivity study on the influences of environmental factors and atmospheric parameters on LST and its temporal variations over bare surfaces, the results indicated that two LST temporal variables, *TN* (the increasing rate of LST normalized by the difference in the NSSR 1.5 and 4.5 h after sunrise in the mid-morning) and td (the time at which the daily maximum temperature occurs) are highly related to the SM [158]. A simple multi-linear model was used to estimate the SM by using the TN and t_d_. The equation is expressed as follows:

(17) $$SWC=b_{0}+b_{1}*\text{ln}(TN*20)+b_{2}*\frac{t_{d}-12}{D_{T}}$$

where SM is the surface soil moisture. The parameters td, *TN* and *D_T_* are calculated from the Noah LSM simulation results, and b_0_, b_1_ and b_2_ are the regression coefficients. The developed model can by used to estimate SM with a high accuracy for a given day (the RMSE is approximately 0.025 m^3^/m^3^).

**Figure 5:**
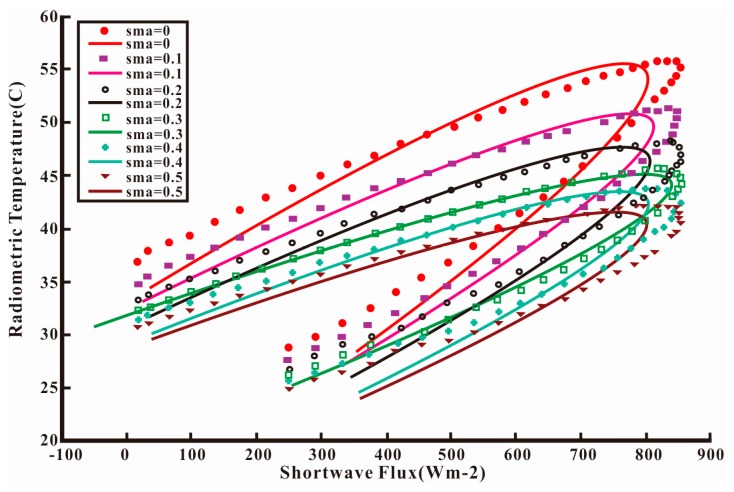
The ellipse change with the different soil moisture content.

Based on the study of Zhao, Song and Leng [159] established a physical elliptical model with 5 coefficients and evaluated the effects of model parameters on the developed ellipse model (Figure 5). A stepwise multi-linear regression was used to fit the SM, and a multi-linear relationship was used to retrieve the daily average SM as follows:

(18) $$SWC=n_{1}*x_{0}+n_{2}*y_{0}+n_{3}*a+n_{4}*\theta +n_{0}$$

where SM is the daily average soil moisture content (m^3^/m^3^) and *x_0_*, *y_0_*, a, and *θ* are the ellipse parameters that represent the center horizontal coordinate and center vertical coordinates of the ellipse and the semi-major axis and rotation angle, respectively. In addition, *n_i_* (*i* = 0, 1, 2, 3, 4) represents the fitting coefficients. When validated by two AmeriFlux sites, the estimation accuracies can reach *R*^2^ = 0.548 when RMSE = 0.088 m^3^ /m^3^ and *R*^2^ = 0.445 when RMSE = 0.126 m^3^ /m^3^. These results demonstrated that the developed model can accurately estimate the daily average SM.

The temporal information-based method relies on the temporal changes of NSSR and LST to establish a relationship with the SM. The elliptical model parameters were used to determine the SM. Accurate estimation results were achieved for specific atmospheric conditions. The main advantages of this method include the following: (1) the proposed model is independent on the soil types; (2) the quantitative SM is determined by using the method rather than from the empirical relationship between SM and the remotely sensed parameters; and (3) the diurnal changes of the land surface parameters are used rather than the instantaneous parameters that have many uncertainties at some moments. However, the limitations of the proposed model are discussed below. (1) The model is suitable for bare soils on cloudless days and the accuracy is greatly reduced in the vegetated areas. This reduction in accuracy occurs because the elliptical shape constructed by the NSSR and LST appears under bare soil conditions. When the surface is covered by vegetation, the surface energy distribution is more complex than that of the bare soil and the elliptical shape is affected by the vegetation interference; (2) the coefficients of the proposed model change with atmospheric conditions. Thus, it is difficult to fix to the coefficients to establish a universal equation for estimating SM. Additionally, how to acquire these model parameters at the regional scale must be addressed before applying the proposed SM model to remotely sensed data [156]. Therefore, these two problems are important issues that concern the application of the model with remotely sensed observations and are promising as a foundation for future work. Moreover, investigating the relationships between the model parameters and atmospheric conditions is probably a promising avenue for making the SM retrieval model universal.

## 5. Current Problems and Discussions

Despite significant progress made since the 1960s in SM retrieval methods, from empirical simplified equations to more complex physically based models using remote sensing technology, and to estimate regional SM and resulting in many SM missions (as shown in Table 4), many problems have still not been solved. These problems are mainly associated with the input parameters of SM models, the estimation accuracy and the physical interpretation of different surface variables from satellite data at the temporal and spatial data/model scales, and the validation of SM obtained from remotely sensed data at the regional/global scale. These issues will be discussed briefly below.

### 5.1. The Uncertainties of the Input Parameters Used in Soil Moisture Estimation Models

The accuracy of the estimated SM is influenced by the input variables that must be accurately obtained from in situ measurements or remote sensing. Inaccurate input parameters can induce many uncertainties in soil moisture estimations. For example, one advantage of the model proposed by Xue and Cracknell [182] is to apply the variations in the surface temperature phase to estimate thermal inertia. However, in the derivation process, the equation requires a priori knowledge of the time at which the maximum LST occurs. In practical applications, authors always use the maximum air temperature observed at ground meteorological stations instead, since the maximum air temperature is easy to obtain. In addition, according to previous studies, the maximum LST is lower than the maximum air temperature by up to 4–5 °C [155]. Thus, accurate maximum air temperature estimation using the remotely sensed data provides an effective means to obtain precise real thermal inertia. In addition, based on the model proposed by Xue, Sobrino et al. developed a new remotely sensed model, the FTA model, to estimate thermal inertia by using the phase differences between the different overpass times of the NOAA satellite. Current problems include: (1) the polar-orbiting satellite data cannot satisfy the requirements of high quality images; and (2) the observed surface times are 2:30, 7:30, 14:30 and 19:30, respectively, and the surface difference is obtained at 2:30 and 14:30. The phase differences are determined at 7:30, 14:30 and 19:30. In addition, with the development of the satellites, the geostationary meteorological satellite can provide many data in one day, which can compensate for the limitations of polar-orbiting satellites. Moreover, large errors can be induced by the LSTs twice each day to simulate the daily LST variations, and more data are required to simulate the diurnal variations in surface temperature [183].

The accuracy of the input parameters partially determined the robustness of the SM estimation models. In practical applications, many studies have ignored the limitations of these models or have selected approximate parameters. Consequently, although the soil moisture estimation can satisfy a certain degree of accuracy, they are usually not used correctly. Soil moisture estimation models are obtained using many preconditions and assumptions. Therefore, model selection based on the underlying conditions and the required input parameters is very important. It is worth noting that the mismatch in scale between the point-based observations and the remotely sensed data remains a problem to be solved.

### 5.2. Soil Moisture under Vegetation

The sensors receive the composite signal from the surface, which includes the soil and vegetation. In the vegetated regions, removing the vegetation effect to estimate SM becomes a problem. Currently, one-source models consider the soil and vegetation as a composite leaf that is called the big leaf model. These models cannot separate the soil from the mixed remotely sensed pixels. Thus, the SM in the vegetated area is manifested by the vegetation index or temperature vegetation dryness index. However, the quantitative SM is not easy to be obtained under vegetation cover. To solve the above-mentioned problems, two-source models (such as ALEXI, N95 et al.) were used to separate the soil and vegetation information and describe the energy exchange among the soil, vegetation and atmosphere. The SM may be accurately estimated if pure soil information can be separated from the composite context.

Additionally, because many soil physical parameters cannot be obtained by using remotely sensed data, this method only can use empirical equations or in situ measurements to determine these variables. Soil texture information exhibits considerable heterogeneity and is difficult to obtain for large regions. In addition, it is difficult to establish a universal relationship for SM across different study areas. To simplify the physical formula and facilitate calculation, the soil physical variables can be parameterized to establish a correlation with the obtained variables from remote sensing. This method may be ultimately a robust method that not only considers the soil physical properties but also can be realized using remote sensing techniques.

### 5.3. Uncertain Quantitative Relationships between the Remotely Sensed Indices/Thermal Inertia and SM

No determinate quantitative relationships between the thermal inertia/drought indices and SM are widely accepted by all the experts. However, many authors have considered this linear relationship as a simple method for determining SM. Several authors have indicated that these variables are not linearly related in empirical statistical experiments. Currently, the commonly applied equations are linear or exponential. However, although the SM estimation methods have matured and the thermal inertia/drought indices can be determined relatively accurately, determining the SM from thermal inertia/drought indices remains uncertain. Thus, a universal and quantitative relationship between the SM and thermal inertia/drought indices must be accurately determined.

### 5.4. Lack of Surface Data for Validating Remotely Sensed SM

Remotely sensed soil moisture validation is a problem in SM estimation research. The most commonly used methods are validated by single point-based or multiple point-based measurements. In addition, many researchers have attempted to use up-scaling or down-scaling methods to validate SM estimates from remote sensing data [184]. Remotely sensed data have wide spatial coverage of the underlying surface, such as 30 m, 1 km, or even several kilometers. In addition, the retrieved SM is the pixel value of the satellite data that indicates the situation of the entire region. However, surface measurements are always conducted at single points and cannot represent the entire region due to the spatial and temporal changes of the SM [185]. Remotely sensed SMs are frequently validated by many measured point values in study areas, which results in many uncertainties. Moreover, the developed SM indices serve as proxies that indicate drought conditions and are not true SM contents. To obtain the actual SM, the wilting and saturation points of the soil in the study area must be known, which can result in errors. Therefore, a surface-to-surface validation method should be developed to solve this problem. Aerial photography or UAV may provide the actual state of the ground surface, which could be used to verify the derived SM.

### 5.5. Uncertainties in the Application of Soil Moisture Estimation Models

As described above, each soil moisture estimation model is developed with preconditions and assumptions. Many uncertainties are introduced by key processes when applying soil moisture estimation models. For example, the spatial-information based method includes two important processes, determining the limiting edge and interpolation between the dry and wet edges. First, determining the dry and wet edges is important for estimating soil moisture from the LST-VI space, which directly affects the retrieval accuracy. In practice, most studies use the least squares method to directly fit scattering at the dry and wet edge. However, due to underlying heterogeneity and different atmospheric forcing, the extracted wet and dry edges are unstable and introduce uncertainties, which eventually lead to SM estimation errors. Currently, two main methods are used to derive the dry and wet edges from scattering in the feature space, the auto-determined edge algorithm and the theoretical edge determination method. The main advantages of the auto-determined edge method are that it is simple to operate, highly accurate, and has wide application. However, one limitation is that the auto-determined edges are changeable with the size of the study area and that the extreme dry and wet points must all be present in the LST-VI space. Sometimes, true dry or wet edges do not exist because energy and water balance must be met under extreme conditions. Thus, many experts have tried to derive theoretical edges based on the surface energy balance principle as a boundary condition. The auto-determined edge method can be used to estimate soil moisture from remote sensing data but results in large errors due to its subjectivity and the effects of atmospheric conditions. The surface energy balance method for solving theoretical edges has a robust physical basis because the requirement for more underlying surface auxiliary data results in a complicated calculation process. It is difficult to obtain pure remote sensing data, and many parameters introduce uncertainty. Therefore, a simple and applicable method for remote sensing to obtain theoretical dry and wet edges is a promising approach. The two-source models can provide wizards for solving this problem that can separate the soil and vegetation surface temperatures. This method can be used to obtain a true dry point for bare soil and full vegetation before deriving theoretically limiting edges by using an energy balance model. Second, many authors have found that LST and SM are negatively correlated in the LST-VI space; thus, numerous studies have been based on the assumption that surface temperature and soil moisture vary linearly with changes in vegetation cover [141]. However, Carlson et al. [186] observed that this change is not linear according to the SVAT model simulation. In practical applications, most scholars still assume that the soil moisture varies linearly in the scatter space. The interpolation of the feature space represents the relationships among SM, LST and FVC. The linear interpolation is simple and easy to operate but cannot truly reflect the SM variations in the feature space [187]. Although the non-linear interpolation is complex and difficult to operate, it is highly accurate [188]. Thus, how can we develop a method to interpolate the space based on a physical mechanism and the SM? Thermal inertia is a physical property of the substance itself. Thus, if thermal inertia is too complicated to use, the ATI could be used to replace it. The ATI may be helpful for interpolating the feature space in future research.

In summary, comprehensive analyses of the aforementioned SM estimation methods based on optical and thermal remote sensing were performed. Although remote multi-monitoring and inversion methods involve different types of fields and relatively broad scopes, they have many shortcomings. In general, despite their susceptibility to clouds, vegetation effects and limited penetration, optical and thermal remote sensing data have high spatial resolutions with multi-satellite sensors and large spatial coverage. Moreover, soil moisture and surface temperatures are strongly correlated. Thus, soil moisture estimations are more promising. Although some models are relatively simple and have clear physical meaning, some methods, such as thermal inertia and apparent thermal inertia methods, are mainly suitable for bare soils or for early crop growth conditions. In addition, due to the impacts of soil texture and type, the relationships between thermal inertia and soil moisture are often nonlinear. However, the method is derived under certain assumptions, and the effects of these assumptions on the retrieval results must be further validated. The triangle/trapezoid method mainly uses the spatial distribution of pixel information when the requirements of study area are large enough and contain a variety of ground conditions, including different vegetation coverage and soil moisture. In some cases, this condition is not easily satisfied. The temporal information-based methods require observation data for long time series from remote sensing on cloudy days. Additionally, the simulated model accuracy and subjective cognitive and empirical descriptions for the study area affect the inversion results. Due to existing problems, some recommendations or solutions may provide references for SM estimations, as described below.

## 6. Conclusions and Perspective

With the problems pointed out above, this paper proposes our recommendation and potential solution in SM estimations.

### 6.1. Combining Optical and Microwave Remote Sensing to Estimate SM

Visible and thermal infrared remote sensing has fine spatial resolutions and broad coverage. The primary differences between these methods are the use of the electromagnetic spectrum and the source of the electromagnetic energy [84]. Visible infrared remote sensing uses characteristic changes in the soil reflectance or vegetation physiology to estimate SM. By contrast, thermal infrared remote sensing uses the responses of soil thermal properties to soil moisture to determine SM. Using integration, the SM may be estimated from soil thermal properties (soil temperature, thermal inertia, etc.) and soil spectrum differences (absorption amplitude, spectrum combination, etc.). In addition, microwave remote sensing is less disturbed by the bad weather or rain. However, it can be easily perturbed by vegetation, and optical and thermal remote sensing can be helpful for removing vegetation disturbances. Therefore, the combination of optical and thermal and microwave remote sensing may have broad application prospects. Additionally, microwave remote sensing can obtain data all-time and all-weather, which can provide great help for soil moisture products over long time series, especially in humid tropical regions.

Moreover, the combination of the thermal inertia method and the LST-NDVI space method may provide a robust method for estimating the SM. Thermal inertia is a soil physical property with soil moisture, and the LST-NDVI method can restrict changes in soil moisture in a specific space. This combination has a strong physical basis, is easy to operate, and may be promising.

### 6.2. The Development of Comparable Soil Moisture Indices

Currently, many drought indices are based on instantaneous remotely sensed data that cannot be compared with other data because of the influences of different atmospheric conditions. For visible infrared remote sensing, long time-series remote sensing data are difficult to obtain and the reference standards are not properly determined. For thermal infrared remote sensing, the derived indices can represent the drought conditions of the study area for a specific time but cannot be compared with each other day by day. The driest and wettest points may not exist in the study area. Thus, determining the dry and wet edge is somewhat subjective and results in errors in the soil moisture estimations. For this problem, the normalization method can be used to normalize the indices to a uniform reference or statistical data can be used to standardize the indices. Only the drought indices from a uniform reference can be physically compared with each other and accurately reflect the true soil moisture conditions.

### 6.3. The Measured Surface Data for True Validation

True validation is the process of assessing the uncertainty of the data products derived independently from the system outputs for the same scale, time and location. Without validation, any methods, models, algorithms, and parameters derived from remotely sensed data cannot be used with confidence [189,190]. True validation must be conducted because validation can help understand the uncertainties of the remotely sensed data, the errors in soil moisture inversion models and uncertainty in the retrieved land surface variables.

Currently, the estimated soil moisture is mostly validated by point-based measurements. The surface SWC data derived from remote sensing are only validated by one or several points’ data measured using techniques. The scale differences can induce large errors in soil moisture retrieval outputs and methods. So, a surface to surface or pixel to pixel validation method should be developed to deal with this problem.

### 6.4. Improvements to the Soil Moisture Estimation Theory

The physical principles of many SM estimation methods include energy balance equations that serve as boundary conditions. According to previous studies, the energy balance is not always closed. Thus, it is relatively easy to induce uncertainties. Moreover, the SM is an important link in the water cycle and the water balance for a specific region should be considered. In addition, the laws and theories regarding soil hydrothermal characteristic can be combined with remotely sensed parameters to reveal the interaction mechanisms of soil moisture. To accurately estimate the SM, the energy and water balance should be considered as well as the soil hydrothermal properties.

Therefore, several limitations exist in the current use of optical and thermal remote sensing SM estimation methods. These methods are based on polar-orbiting earth observation satellite advances, and polar-orbiting satellites usually obtain three or four ground observations each day. Lacking data sources limits the development of soil moisture retrieval methods. Fortunately, the geostationary meteorological satellites can achieve high frequency and continuous ground observations and high temporal resolution data that polar-orbiting satellites cannot do. Geostationary meteorological satellites can provide favorable conditions and opportunities for estimating surface soil moisture [191,192,193,194,195,196,197,198,199,200,201,202,203].

## Figures and Tables

**Table 1:** Remotely sensed methods used in the soil moisture estimation study [62].

Category	Methods	Advantages	Disadvantages	References
Optical	Visible-based methods	Good spatial resolution, multi-bands available, mature technology	Vegetation interference, night effects and poor temporal resolution	[63,64]
	Thermal Infrared-based methods	Good spatial resolution, multiple satellites available	Vegetation interference, cloudy contamination, night effects, poor temporal resolution and atmospheric effects	[65,66]
Passive microwave	(semi-)empirical, physically-based methods	High accuracy for bare soil surfaces, unlimited by clouds and/or daytime conditions, high temporal resolution	Coarse spatial resolution, influenced by vegetation cover and surface roughness	[67,68]
Active microwave	(semi-)empirical, physically-based methods	Fine spatial resolution, unlimited by clouds and/or daytime conditions	influenced by surface roughness & vegetation cover amount, coarse temporal resolution	[69,70,71]
Synergistic methods	Optical & Thermal Infrared	High spatial resolution, simple & straightforward implementation	limited to cloud-free &daytime conditions, poor temporal resolution, low penetration depth	[72,73]
	Active & passive MW	improved temporal and spatial resolution	SMC scaling & validation needs caution, different SMC measurement depths	[74,75]
	MW & optical	Minimized vegetation and surface roughness effects	SMC scaling & validation needs caution, different SMC measurement depths	[76]

**Table 2:** Comparison of several common drought indices.

Name	Equations	Advantages	Disadvantages	References
VCI	(2)	Removing weather and site effects	Difficult to obtain data sources are and error and volatility of instantaneous vegetation index	[100,101]
AVI	(3)	Reference standards and considering weather effect	Subjectivity andno annual variation	[102]
NDWI	(4)	More sensitive to SM and insensitive to atmospheric conditions	Limitations in vegetated areas	[103]
NMDI	(5)	Quick response to moisture changes	The mixed pixel of vegetation and soil	[104,105]
PDI	(6)	Suitable for bare soil	Limited in vegetated areas and non-flat regions of different soil types.	[107]
MPDI	(7)	Consideration of vegetation influence	Invariant soil color and fixed soil line	[108]

**Table 3:** Comparisons of the common thermal inertia methods.

Methods	Principle	Advantages	Limitations	References
The physical basis analytical method	Solving the one-dimensional equation by the boundary conditions	Robust physical principle	More auxiliary data and complex calculation	[109,110,111,112,113,114,115,116,117,118]
The model based on the amplitude and phase information of LST	The phase and amplitude information are used to solve the boundary conditions	Easy and simple to operate, less ground-based measurement data	More approximations and complicate solving process	[119,120,121,122,123,124,125]
Analysis method based on energy sources	The soil heat flux is the source of thermal inertia	Less input parameters and simple calculation	High-demand conditions, coarse images at night	[126,127,128,129]
Remote sensing methods combined with soil physical parameters	The definition of thermal inertia	Clear physical meaning	the requirement of the soil physical parameters	[130,131,132]

**Table 4:** Currently available soil moisture products by remotely sensors.

Sensors/Missions	Characteristics	Advantages	Limitations	References
SMAP	1.41 GHz, H, V and HV or VH, IFOV: 40 × 40 km, Swath width: 1000 km, 3 days	high-resolution, high-accurate soil moisture, corrections for rotation	highly influenced by surface roughness, vegetation canopy structure and water content	[160,161,162,163]
SMOS	1.4 GHz, H and V, IFOV: 43 × 43 km, 3 days	multi-angular acquisition capability, low sensitivity to cloud and vegetation contamination, high sensitivity to soil moisture fluctuations	poor spatial resolutions, highly influenced by surface roughness and vegetation cover	[164,165,166,167,168,169]
AMSR-E	6.6, 10.65, 18.7, 23.8, 36.5, 89GHz, H and V, IFOV: 76 × 44, 49 × 28, 28 × 16, 31 × 18, 14 × 8, 6 × 4 km, Swath width: 1445 km, 2 days	Long-term observations, high revisit frequency	coarse-scale resolution, data records overlap, small penetration depth	[170,171,172,173,174]
Sentinel-1	5.405 GHz, HH-HV and VV-VH, 3 h or less	High-accurate soil moisture, high spatial and temporal resolution	highly influenced by surface roughness and vegetation conditions	[175,176,177]
Landsat	30 m (15 m for Band 8 of OLI), 16 days	Good spatial resolution, multi-bands available	Vegetation and cloud interference, night effects	[178,179]
MODIS	1000 m (250 m for panchromatic bands), 1 day	Good spatial resolution, multiple satellites available	Vegetation interference, cloudy contamination, night and atmospheric effects	[180,181]
